# Sex determination in skeletal remains from the medieval Eastern Adriatic coast – discriminant function analysis of humeri

**DOI:** 10.3325/cmj.2013.54.272

**Published:** 2013-06

**Authors:** Željana Bašić, Ivana Anterić, Katarina Vilović, Anja Petaros, Alan Bosnar, Tomislav Madžar, Ozren Polašek, Šimun Anđelinović

**Affiliations:** 1 University Department for Forensic Sciences, University of Split, Split, Croatia; 2 University of Split School of Medicine, Split, Croatia; 3 Department of Forensic Medicine and Criminalistics, University of Rijeka School of Medicine, Rijeka, Croatia; 4 University of Zagreb School of Medicine, Zagreb, Croatia

## Abstract

**Aim:**

To investigate the usefulness of humerus measurement for sex determination in a sample of medieval skeletons from the Eastern Adriatic Coast. Additional aim was to compare the results with contemporary female population.

**Methods:**

Five humerus measurements (maximum length, epicondylar width, maximum vertical diameter of the head, maximum and minimum diameter of the humerus at midshaft) for 80 male and 35 female medieval and 19 female contemporary humeri were recorded. Only sufficiently preserved skeletons and those with no obvious pathological or traumatic changes that could affect the measurements were included. For ten samples, analysis of DNA was performed in order to determine sex using amelogenin.

**Results:**

The initial comparison of men and women indicated significant differences in all five measures (*P* < 0.001). Discriminant function for sex determination indicated that as much as 85% of cases could be properly categorized, with better results in men (86%) than women (80%). Furthermore, the comparison of the medieval and contemporary women did not show significant difference in any of the measured features. Sex results obtained by anthropological and DNA analysis matched in all 10 cases.

**Conclusion:**

The results indicate that humerus measurement in Croatian medieval population may be sufficient to determine the sex of the skeleton. Furthermore, it seems that secular changes have not substantially affected contemporary population, suggesting that the results of this study are transferable to contemporary population as well.

Once the skeletal remains are uncovered, anthropologists initially aim to reconstruct the biological profile of the person, which includes sex, age, and height estimation. During the reconstruction process, numerous issues may arise, including bone fragmentation and poor preservation of skeletal remains, coupled with the complexity of human skeleton (1,2). Sex determination is one of the first and basic steps of assessing the biological profile. Although the analysis of DNA is the most reliable method for sex determination (3), it is also the most expensive and time consuming method, which can also be hindered by local conditions. This may especially be true in cases of poor preservation of the remains, inhibitors effects, or a small amount of extracted DNA from the sample.

In absence of DNA results, skeletal remains can be used to infer subject’s sex via two methods, morphological and anthropometric. The morphological approach is based on the examination of the bones that show the strongest sexual dimorphism, principally the skull and the pelvis (4). However, this method is not always reliable, especially if the skull is fragmented or incomplete. Age can also affect the results, especially in elderly women, in which morphological characteristics of the skull tend to resemble those of men (5). Although morphological methods are very important for a preliminary sex assessment, they additionally rely on the experience of the examiner and are therefore rather subjective and unreliable.

The second approach is based on anthropometric analysis, which relies on the bone measurements. The main analytic approach is based on discriminant function analysis, which attempts to classify subjects into each of the sexes, by using either one or more bones (6). This kind of analysis is a very important quantitative method (7) for sex determination as it reduces the subjectivity of the examiner (2,4,8). So far, only a few such studies have been published using Croatian bone samples. These include medieval and contemporary femurs and tibias (8-11) and medieval and contemporary mandibles and teeth (6,12). Such studies are important, since clear differences were observed in different populations (13-15), making this a locally-specific feature that requires the development of regional standards, applicable for local population (16).

Besides already used femurs and tibias, humerus is another long bone from the body that is presumably informative for sex determination. This idea was initially derived from the empirical investigations of the skeletal remains, and further supported by the previously reported sexual dimorphism of humeri (7,17), even in cases of severe bone fragmentation (18). Furthermore, such location-specific results may be of interest in modern forensics as well, since observed changes in the skeleton marked predominantly by the increase in height (19), appear to be proportional, with no indication of sexual dimorphism in ancient and modern samples (20). Therefore, the aim of this study was to investigate the possibility to determine the sex of the subject based on anthropometric analysis of humeri measures.

## Materials and methods

We analyzed a sample of 80 male and 35 female humeri from 7 medieval sites from eastern Adriatic coast: Svećurje -Žestinj (dated in 9/11th century) (21), Rižinice (dated 9/10th century), Bijaći Stombrate (dated in 9/10th century) (22), Ostrovica Greblje (dated in 9th century) (23), Šopot Benkovac (dated in 14/15th century), Kamenmost Kaldrma (dated in 14/15th century) (24), and Otok Vuletina rupa – Grebčine (dated 17/18th century) (25). These graveyards were shown by the archeological excavations to be of typical Croatian culture, creating a relatively homogenous group of subjects involved in this analysis.

In order to compare these results to the contemporary population, we additionally used another sample of skeletons, dated to the end 19th century and beginning of the 20th century. This sample consisted of 19 female skeletons from the Kozala monastery graveyard (Rijeka, Croatia). The validation sample selection was based on the available well preserved skeletons. In both samples, only those with overall very good preserved status were included, with the subject sex determined using standard anthropological methods for sex determination. Sex was determined by examination of sex specific pelvis and skull morphological characteristics. On the pelvis we examined the greater sciatic notch, pelvis size and shape, ventral arc, subpubic concavity, and medial aspect of ischiopubic ramus, and on the skull: nuchal crest, mastoid process, supraorbital margin and ridge, mental eminence, and ramal flexion (4,26-28). Only skeletons meeting the criteria of full confidence in sex determination were included in the study. In turn, all of the samples where substantial damage was recorded, samples with obvious pathological findings, traumas, and deformations that can alter the bone structure or affect the measurements were excluded from the analysis. A total of 459 medieval adult skeletons were analyzed, 115 of which met the described criteria, while out of 47 contemporary female skeletons, 19 met the described criteria.

A total of five measures were made:

1. maximum length of the humerus – the direct distance from the most superior point on the head of the humerus to the most inferior point on the trochlea,

2. epicondylar width of the humerus – the distance of the most laterally protruding point on the lateral epicondyle from the corresponding projection of the medial epicondyle,

3. maximum vertical diameter of the head of the humerus – the direct distance between the most superior and inferior points on the border of the articular surface,

4. maximum diameter of the humerus at midshaft – the maximum diameter of the midshaft measured by turning the bone until the maximum diameter is obtained,

5. minimum diameter of the humerus at midshaft – the least diameter of the midshaft (29).

The measurements were taken using osteometric board and sliding caliper. Two measurements were performed independently by two authors and later compared. In case of discrepancies between the measured values the measurement was repeated. Measurements were taken from the left side whenever possible.

### Analysis of DNA

In addition, 10 tooth samples from 10 different individuals from the excavation site Otok Vuletina rupa – Grebčine were taken for additional confirmation that morphometric sex determination is 98% reliable when both pelvis and skull are available (4). For these 10 samples, sex was determined by analysis of the amelogenin gene using protocols described previously (30,31). Contamination of DNA was prevented using previously described protocol (32). Teeth were washed with distilled water and dried. Using circular saw type 900 (KaVo Elektrotechnisches Werk, Vertriebsgesellschaft GmbH, Leutkirch, Germany), the teeth surface and canals were removed around 2-3 mm in depth. The sawdust was washed with distilled water, and the teeth were cleaned with 5% commercial bleach. Using liquid nitrogen the teeth were frozen and then ground into powder in a cylinder crushing tool. For each sample, 1 g of teeth powder was used and cleaned with EDTA for 5 days. DNA was extracted using the 3 mL extraction buffer (10 μmol/L Tris, pH 8.0; 100 μmol/L NaCl; 50 μmol/L EDTA, pH 8.0; and 0.5% sodium dodecyl sulfate, SDS) and 150 μL of 20 mg/mL proteinase K (PK) and incubated for 24-48 hours at 56°C with shaking. The liquefied teeth were rinsed with Phenol: Chloroform: Isoamyl Alcohol (25:24:1) and centrifuged twice for 10 minutes at 5000 RPM. The top layer of liquefied teeth was placed in a new 15-mL tube to be mixed with 3 mL n-butanol and centrifuged for 10 minutes at 5000RPM. The bottom layer was transferred to an Amicon tube and repeatedly washed and centrifuged with ddH2O for 10 minutes at 2600RPM (at least twice). Amplification was performed using AmpFlSTR® MiniFiler PCR Amplification Kit (Applied Biosystems, Foster City, CA, USA) and polymerase chain reaction (PCR) was performed in GeneAmp PCR System 9700 (Applied Biosystems). PCR products were typed on ABI Prism 310 Genetic Analyzer (Applied Biosystems).

### Statistical analysis

Descriptive statistics encompassed numbers and percentages for categorical variables, coupled with means and standard deviations for numerical variables. Inferential statistics was based on an independent *t* test used for analysis of sex-related differences in measured variables. Discriminant analysis was used to define the existence of sex-discriminatory variables, with calculation of percent of correctly classified cases as the validation measure. In line with previous analyses, we also employed a regression analysis, which aimed to identify sex-related differences, in line with similar previous studies (6,8,10,11,33). All analyses were performed using SPSS (ver 18; SPSS Inc, Chicago, IL, USA), with the significance level set at *P* < 0.05.

## Results

This study was based on 115 bone samples, 80 male and 35 female, from 7 medieval locations (Table 1). Most of the analyzed medieval humeri were from the left side (80 samples: 26 women, 54 men), while a smaller portion was from the right side (35 samples: 9 women, 24 men). Of the contemporary female samples, 19 were left humeri. The comparison of the left and right humeri did not yield significant difference for any of the measured features (data not shown).

**Table 1:** The number of humeri from each excavation site

Site	Women	Men	Total
Svećurje	3	5	8
Rižinice	1	1	2
Bijaći Stombrate	7	10	17
Ostrovica Greblje	10	25	35
Šopot Benkovac	2	10	12
Kamenmost Kaldrma	7	10	17
Otok Vuletina rupa – Grebčine	5	19	24
Total	35	80	115

Since all of them were of comparable bone status, we performed a direct comparison, which suggested that all five humerus measures in the medieval period were significantly different between men and women (Table 2).

**Table 2:** Initial comparison of the humeri measurement in men and women from medieval period (mean ± standard deviation)

Measurement; mm	Men (n = 80)	Women (n = 35)	Index	*P*
Maximum length	327.92 ± 19.05	304.24 ± 17.79	107.78	<0.001
Epicondilar width	63.53 ± 4.55	56.74 ± 3.60	111.97	<0.001
Maximum vertical head diameter	46.86 ± 3.65	41.65 ± 2.04	112.51	<0.001
Maximum diameter at midshaft	23.38 ± 1.96	21.10 ± 1.45	110.81	<0.001
Minimum diameter at midshaft	19.60 ± 2.25	16.92 ± 1.64	115.84	<0.001

The main step of the analysis involved discriminatory function, which suggested that the humeri were more effective in sex determination of men than women (Table 3). In total, 84.8% of the subjects were correctly classified using this function. Additionally, we aimed to see if there were fixed combinations of the measurement that would provide better sex determination functions. When maximum and minimum head diameters were used in discrimination analysis, the results indicated that 75.2% of cases were correctly classified. In turn, the use of head width and epycondilar width suggested that 82.5% of the subjects were correctly classified, thus suggesting that all five measurements provided the most informative sex determination set.

**Table 3:** Accuracy for sex determination functions for medieval period (mean ± standard deviation)

Measurement; mm	Men	Women
All variables	58 ± 86.57	20 ± 80.00
Maximum length	69 ± 90.79	17 ± 50.00
Epicondilar width	65 ± 87.84	19 ± 65.52
Maximum vertical head diameter	62 ± 84.93	24 ± 72.73
Maximum diameter at midshaft	75 ± 93.75	11 ± 50.00
Minimum diameter at midshaft	71 ± 88.75	18 ± 52.94

Furthermore, we aimed to see if it was possible to define the range of measurements which provided *a-priori* correct classification for men and women. The results suggested that the majority of the measures had a wide range of overlap, with the worst result for maximum head diameter, which seemed to have the greatest overlap between men and women (Table 4).

**Table 4:** Overlap ranges of the analyzed measurements for men and women

Measurement; mm	Sex range		
	female	unclear	male
Maximum length	<247	247-385	>385
Epicondilar width	<53	53-65	>65
Maximum vertical head diameter	-	38-46	>46
Maximum diameter at midshaft	<20	20-23	>23
Minimum diameter at midshaft	<16	16-20	>20

Lastly, we aimed to compare the medieval and contemporary samples. This analysis suggested that neither of the analyzed measures were significantly different between the two subsamples (Table 5).

**Table 5:** Comparison of the medieval and contemporary women (mean ± standard deviation)

Measurement; mm	Medieval women (n = 35)	Contemporary women (n = 19)	*P*
Maximum length	304.24 ± 17.79	303.14 ± 18.62	0.869
Epicondilar width	56.74 ± 3.60	56.15 ± 4.98	0.637
Maximum vertical head diameter	41.65 ± 2.04	41.57 ± 2.83	0.984
Maximum diameter at midshaft	21.10 ± 1.45	20.38 ± 2.04	0.160
Minimum diameter at midshaft	16.92 ± 1.64	17.23 ± 2.13	0.369

Sex was determined by amelogenin analysis for 10 skeletons, 9 male and 1 female. Sex results obtained by DNA and morphometric anthropological analysis matched in all 10 cases.

## Discussion

The results of this study show that humeral measurements of the medieval Croatian population may serve as the reasonably good estimate of sex. As humeri had not before been analyzed in the Croatian population for this purposes, the main aim of this study was to test if the humeral measurements were a reliable sex indicator. Determination of sex is the first step in determination of biological profile of a person, that is, the first step in individualization, and in forensic sciences – identification of an individual. As the morphological method of sex determination is subjective and relies mostly on the experience of the examiner, anthropometric methods have been developed. These methods include discriminant functions for sex determination for almost every bone in human body. But, as reported by various authors, these functions tend to be population-specific, therefore, the imperative of every region is to develop its own functions (13-16).

We obtained better classification results for men, and the overall pattern suggested that measurements of the entire humerus provided the best determination possibilities, somewhat better than the isolated central parts of humerus or its proximal fragments. These findings are largely in line with previous studies (7,17,30), which have also pointed out to the population-specific estimates (13-16).

When using one function, the most accurate function for women is the maximum head diameter (which classifies correctly 72.73% of women), while for men it is the maximal diameter at midshaft (which classifies correctly 93.75%of men). It is interesting that the largest gap between men and women is visible in both of these functions: the most accurate function for male sex determination is also the most inaccurate function for female sex determination and vice versa. This can be the result of variability between sexes, but a difference in a sample size has also to be considered.

Prediction of sex is of higher accuracy in men, which was supported by other authors (34).

The sex difference in the humeral measurements are probably due to differential bone remodeling between sexes, in men cortical bone develops more during adolescence (35).

The two measurements with greatest sex difference are the maximum length and the maximum diameter at midshaft, which was also found by other authors (36). Some authors believe that this is common in populations with extremely high or extremely low protein consumption (37), while other suggest that the circumferential measurements are more important for sex determination because of the influence of physical activity on bone (38). Some authors found that the most effective single dimensions were vertical head diameter (18,39) and epicondylar breadth (40), which indicates the necessity of developing regional sex discriminant functions.

One of the interesting findings is the similarity of the humeral measurements for medieval and contemporary population. This result suggests that any secular changes that were happening over time did not affect humeri. An important conclusion that can be drawn from this study is the possibility to use the results inter-changeably in both medieval and contemporary populations. This finding is very interesting, since similar studies performed on femurs and tibias showed the opposite result (8-11). Both of these conclusions can be joined into a more general result, suggesting that some parts of the skeleton were seemingly more affected than others. An analysis of contemporary male humeral samples is necessary to see if the male sample will follow the same trend. This is important because if the residence is patrilocal, the variability between male skeletons will be small inside one cemetery, but considerable between various cemeteries. On the other hand, female skeletons will vary more inside one cemetery than between cemeteries (41).

There are several study limitations. First, the overall sample size was relatively small, especially the contemporary population. The analyzed medieval sample encompassed a long stretch of time and probably a diverse set of environmental conditions, which could have affected the final result. The results reported here are furthermore limited only to well-preserved skeletons, without any substantial skeletal changes that could affect the results.

Our findings, showing homogeneity and lack of significant differences between medieval and contemporary populations are in line with some of the previous studies, which suggested homogeneity of the population over time, based on the Y chromosome (42) and mitochondrial DNA (43). Such findings are at the moment based on limited amount of evidence, suggesting that future studies should aim to encompass larger periods of time and more precisely defined population in order to establish a solid understanding of the anthropometric and genetic changes in the population of contemporary Croatia.
